# Supplementary material for: The Germination and Growth of Two Strains of Bacillus cereus in Selected Hot Dishes After Cooking
Source: Foods. 2025 Jan 9;14(2):194. doi: 10.3390/foods14020194 (PMC11764521; doi:10.3390/foods14020194)
Supplement: Supplementary file 1 [file foods-14-00194-s001.zip › Kameník et al_2024_Supplementary File S1.pdf]

Supplementary File S1. Preparation method of the hot ready-to eat mashed potatoes, mushroom sauce, tomato sauce, and cooked rice according to the recipes by Runštuk et al. [18]

Mashed potatoes

Peel the potatoes, wash them, cut them into quarters and boil them in salted water for 25 minutes. Drain the water, mix them in a 3L FC3SP blender (supplied by Maso-profit, s.r.o., Prague, Czech Republic) and add hot milk (50 °C) until a smooth mash is obtained. Heat the mash to 80 °C before the next experiment. For 2 kg of potatoes, add 0.5 l of cow's milk (1.5% fat), add salt to 1%.

Mushroom sauce

Prepare a darker roux and boil in water. Sauté chopped wild mushrooms in butter with onion, add to sauce and bring to a boil. Blend and heat to 80 °C. Add salt to 1%.

Tomato sauce

Fry the chopped onion in fat, add the chopped carrots, parsley and celery and fry. Pour in the beef broth, add the roux and tomatoes. Bring to a boil, blend and heat to 80 °C. Add salt to 1%.

Cooked rice

Wash long-grain peeled rice with water, add water (0.5 l of water per 400 g of rice) and cook until soft (the water evaporates). Add salt to 1%.
