# Supplementary material for: The Germination and Growth of Two Strains of Bacillus cereus in Selected Hot Dishes After Cooking
Source: Foods. 2025 Jan 9;14(2):194. doi: 10.3390/foods14020194 (PMC11764521; doi:10.3390/foods14020194)
Supplement: Supplementary file 1 [file foods-14-00194-s001.zip › Kameník et al_2024_Table S1.pdf]

Table S1. Growth of *Bacillus cereus* in hot dishes

Carbonara						
Temp	Hours	Strain	experiment :	log CFU/g	experiment 2	log CFU/g
40 °C	0 h	BC CCM 869	1700	3,230448921	250	2,397940009
40 °C	0 h	BC DSM 4312	3500	3,544068044	5500	3,740362689
40 °C	0 h	control	25	1,397940009	25	1,397940009
40 °C	0.5 h	BC CCM 869	1500	3,176091259		#ČÍSLO!
40 °C	0.5 h	BC DSM 4312	4800	3,681241237		#ČÍSLO!
40 °C	0.5 h	control	25	1,397940009		#ČÍSLO!
40 °C	1 h	BC CCM 869	1400	3,146128036	50	1,698970004
40 °C	1 h	BC DSM 4312	4900	3,69019608	6100	3,785329835
40 °C	1 h	control	25	1,397940009	25	1,397940009
40 °C	2 h	BC CCM 869	1500	3,176091259	150	2,176091259
40 °C	2 h	BC DSM 4312	12000	4,079181246	52000	4,716003344
40 °C	2 h	control	25	1,397940009	25	1,397940009
40 °C	2.5 h	BC CCM 869		#ČÍSLO!	150	2,176091259
40 °C	2.5 h	BC DSM 4312		#ČÍSLO!	180000	5,255272505
40 °C	2.5 h	control		#ČÍSLO!	25	1,397940009
40 °C	3 h	BC CCM 869	3000	3,477121255	300	2,477121255
40 °C	3 h	BC DSM 4312	160000	5,204119983	440000	5,643452676
40 °C	3 h	control	25	1,397940009	25	1,397940009
40 °C	4 h	BC CCM 869	55000	4,740362689	2500	3,397940009
40 °C	4 h	BC DSM 4312	3000000	6,477121255	7800000	6,892094603
40 °C	4 h	control	25	1,397940009	25	1,397940009
50 °C	0 h	BC CCM 869	1300	3,113943352	25	1,397940009
50 °C	0 h	BC DSM 4312	2400	3,380211242	3600	3,556302501
50 °C	0 h	control	25	1,397940009	25	1,397940009
50 °C	0.5 h	BC CCM 869	1000	3		#ČÍSLO!
50 °C	0.5 h	BC DSM 4312	3100	3,491361694		#ČÍSLO!
50 °C	0.5 h	control	25	1,397940009		#ČÍSLO!
50 °C	1 h	BC CCM 869	1000	3	25	1,397940009
50 °C	1 h	BC DSM 4312	2400	3,380211242	4600	3,662757832
50 °C	1 h	control	25	1,397940009	25	1,397940009
50 °C	2 h	BC CCM 869	1300	3,113943352	25	1,397940009
50 °C	2 h	BC DSM 4312	2500	3,397940009	14000	4,146128036
50 °C	2 h	control	25	1,397940009	25	1,397940009
50 °C	2.5 h	BC CCM 869		#ČÍSLO!	25	1,397940009
50 °C	2.5 h	BC DSM 4312		#ČÍSLO!	35000	4,544068044
50 °C	2.5 h	control		#ČÍSLO!	25	1,397940009
50 °C	3 h	BC CCM 869	860	2,934498451	100	2
50 °C	3 h	BC DSM 4312	2000	3,301029996	63000	4,799340549
50 °C	3 h	control	25	1,397940009	25	1,397940009
50 °C	4 h	BC CCM 869	1400	3,146128036	25	1,397940009
50 °C	4 h	BC DSM 4312	1300	3,113943352	28000	4,447158031
50 °C	4 h	control	25	1,397940009	25	1,397940009
60 °C	0 h	BC CCM 869	1100	3,041392685	25	1,397940009
60 °C	0 h	BC DSM 4312	2300	3,361727836	4100	3,612783857
60 °C	0 h	control	25	1,397940009	25	1,397940009
60 °C	0.5 h	BC CCM 869	1500	3,176091259		#ČÍSLO!
60 °C	0.5 h	BC DSM 4312	1900	3,278753601		#ČÍSLO!
60 °C	0.5 h	control	25	1,397940009		#ČÍSLO!
60 °C	1 h	BC CCM 869	950	2,977723605	50	1,698970004
60 °C	1 h	BC DSM 4312	1100	3,041392685	2500	3,397940009
60 °C	1 h	control	25	1,397940009	25	1,397940009
60 °C	2 h	BC CCM 869	860	2,934498451	25	1,397940009
60 °C	2 h	BC DSM 4312	950	2,977723605	1900	3,278753601
60 °C	2 h	control	25	1,397940009	25	1,397940009
60 °C	2.5 h	BC CCM 869		#ČÍSLO!	25	1,397940009
60 °C	2.5 h	BC DSM 4312		#ČÍSLO!	2100	3,322219295
60 °C	2.5 h	control		#ČÍSLO!	25	1,397940009
60 °C	3 h	BC CCM 869	500	2,698970004	100	2
60 °C	3 h	BC DSM 4312	950	2,977723605	1800	3,255272505
60 °C	3 h	control	25	1,397940009	25	1,397940009
60 °C	4 h	BC CCM 869	680	2,832508913	50	1,698970004
60 °C	4 h	BC DSM 4312	600	2,77815125	2300	3,361727836
60 °C	4 h	control	25	1,397940009	25	1,397940009

RATATOUILLE						
Temp	Hour	Strain	experiment	log CFU/g	experiment 2	log CFU/g
40 °C	0 h	BC CCM 869	300	2,477121255	150	2,176091259
40 °C	0 h	BC DSM 4312	200	2,301029996	1800	3,255272505
40 °C	0 h	bez zaočkování	25	1,397940009	25	1,397940009
40 °C	0.5 h	BC CCM 869	640	2,806179974		#ČÍSLO!
40 °C	0.5 h	BC DSM 4312	250	2,397940009		#ČÍSLO!
40 °C	0.5 h	bez zaočkování	25	1,397940009		#ČÍSLO!
40 °C	1 h	BC CCM 869	590	2,770852012	200	2,301029996
40 °C	1 h	BC DSM 4312	250	2,397940009	1500	3,176091259
40 °C	1 h	bez zaočkování	25	1,397940009	25	1,397940009
40 °C	2 h	BC CCM 869	1000	3	150	2,176091259
40 °C	2 h	BC DSM 4312	300	2,477121255	1300	3,113943352
40 °C	2 h	bez zaočkování	25	1,397940009	25	1,397940009
40 °C	2.5 h	BC CCM 869		#ČÍSLO!	50	1,698970004
40 °C	2.5 h	BC DSM 4312		#ČÍSLO!	2300	3,361727836
40 °C	2.5 h	bez zaočkování		#ČÍSLO!	25	1,397940009
40 °C	3 h	BC CCM 869	550	2,740362689	50	1,698970004
40 °C	3 h	BC DSM 4312	400	2,602059991	1500	3,176091259
40 °C	3 h	bez zaočkování	25	1,397940009	25	1,397940009
40 °C	4 h	BC CCM 869	500	2,698970004	25	1,397940009
40 °C	4 h	BC DSM 4312	200	2,301029996	3800	3,579783597
40 °C	4 h	bez zaočkování	25	1,397940009	25	1,397940009
50 °C	0 h	BC CCM 869	590	2,770852012	450	2,653212514
50 °C	0 h	BC DSM 4312	400	2,602059991	2500	3,397940009
50 °C	0 h	bez zaočkování	25	1,397940009	25	1,397940009
50 °C	0.5 h	BC CCM 869	600	2,77815125		#ČÍSLO!
50 °C	0.5 h	BC DSM 4312	200	2,301029996		#ČÍSLO!
50 °C	0.5 h	bez zaočkování	25	1,397940009		#ČÍSLO!
50 °C	1 h	BC CCM 869	550	2,740362689	50	1,698970004
50 °C	1 h	BC DSM 4312	200	2,301029996	730	2,86332286
50 °C	1 h	bez zaočkování	25	1,397940009	25	1,397940009
50 °C	2 h	BC CCM 869	1000	3	150	2,176091259
50 °C	2 h	BC DSM 4312	250	2,397940009	1300	3,113943352
50 °C	2 h	bez zaočkování	25	1,397940009	25	1,397940009
50 °C	2.5 h	BC CCM 869		#ČÍSLO!	100	2
50 °C	2.5 h	BC DSM 4312		#ČÍSLO!	1200	3,079181246
50 °C	2.5 h	control		#ČÍSLO!	25	1,397940009
50 °C	3 h	BC CCM 869	450	2,653212514	25	1,397940009
50 °C	3 h	BC DSM 4312	450	2,653212514	1200	3,079181246
50 °C	3 h	control	25	1,397940009	25	1,397940009
50 °C	4 h	BC CCM 869	100	2	200	2,301029996
50 °C	4 h	BC DSM 4312	150	2,176091259	1300	3,113943352
50 °C	4 h	control	25	1,397940009	25	1,397940009
60 °C	0 h	BC CCM 869	590	2,770852012	25	1,397940009
60 °C	0 h	BC DSM 4312	250	2,397940009	300	2,477121255
60 °C	0 h	control	25	1,397940009	25	1,397940009
60 °C	0.5 h	BC CCM 869	650	2,812913357		#ČÍSLO!
60 °C	0.5 h	BC DSM 4312	25	1,397940009		#ČÍSLO!
60 °C	0.5 h	control	25	1,397940009		#ČÍSLO!
60 °C	1 h	BC CCM 869	300	2,477121255	100	2
60 °C	1 h	BC DSM 4312	50	1,698970004	250	2,397940009
60 °C	1 h	control	25	1,397940009	25	1,397940009
60 °C	2 h	BC CCM 869	200	2,301029996	50	1,698970004
60 °C	2 h	BC DSM 4312	50	1,698970004	350	2,544068044
60 °C	2 h	control	25	1,397940009	25	1,397940009
60 °C	2.5 h	BC CCM 869		#ČÍSLO!	25	1,397940009
60 °C	2.5 h	BC DSM 4312		#ČÍSLO!	450	2,653212514
60 °C	2.5 h	control		#ČÍSLO!	25	1,397940009
60 °C	3 h	BC CCM 869	350	2,544068044	25	1,397940009
60 °C	3 h	BC DSM 4312	50	1,698970004	150	2,176091259
60 °C	3 h	control	25	1,397940009	25	1,397940009
60 °C	4 h	BC CCM 869	200	2,301029996	25	1,397940009
60 °C	4 h	BC DSM 4312	150	2,176091259	200	2,301029996
60 °C	4 h	control	25	1,397940009	25	1,397940009

Mushroom sauce						
Temp	Hour	Strain	experiment	log CFU/g	experiment	log CFU/g
40 °C	0 h	BC CCM 869	860	2,934498451	150	2,176091259
40 °C	0 h	BC DSM 4312	5300	3,72427587	13000	4,113943352
40 °C	0 h	control	25	1,397940009	25	1,397940009
40 °C	0.5 h	BC CCM 869	1000	3		#ČÍSLO!
40 °C	0.5 h	BC DSM 4312	5400	3,73239376		#ČÍSLO!
40 °C	0.5 h	control	25	1,397940009		#ČÍSLO!
40 °C	1 h	BC CCM 869	1100	3,041392685	300	2,477121255
40 °C	1 h	BC DSM 4312	4300	3,633468456	13000	4,113943352
40 °C	1 h	control	25	1,397940009	25	1,397940009
40 °C	2 h	BC CCM 869	1000	3	200	2,301029996
40 °C	2 h	BC DSM 4312	4800	3,681241237	13000	4,113943352
40 °C	2 h	control	25	1,397940009	25	1,397940009
40 °C	2.5 h	BC CCM 869		#ČÍSLO!	150	2,176091259
40 °C	2.5 h	BC DSM 4312		#ČÍSLO!	14000	4,146128036
40 °C	2.5 h	control		#ČÍSLO!	25	1,397940009
40 °C	3 h	BC CCM 869	500	2,698970004	100	2
40 °C	3 h	BC DSM 4312	22000	4,342422681	28000	4,447158031
40 °C	3 h	control	25	1,397940009	25	1,397940009
40 °C	4 h	BC CCM 869	2100	3,322219295	250	2,397940009
40 °C	4 h	BC DSM 4312	2700000	6,431363764	2800000	6,447158031
40 °C	4 h	control	25	1,397940009	25	1,397940009
50 °C	0 h	BC CCM 869	910	2,959041392	100	2
50 °C	0 h	BC DSM 4312	1500	3,176091259	20000	4,301029996
50 °C	0 h	control	25	1,397940009	25	1,397940009
50 °C	0.5 h	BC CCM 869	700	2,84509804		#ČÍSLO!
50 °C	0.5 h	BC DSM 4312	2000	3,301029996		#ČÍSLO!
50 °C	0.5 h	control	25	1,397940009		#ČÍSLO!
50 °C	1 h	BC CCM 869	1400	3,146128036	150	2,176091259
50 °C	1 h	BC DSM 4312	2400	3,380211242	15000	4,176091259
50 °C	1 h	control	25	1,397940009	25	1,397940009
50 °C	2 h	BC CCM 869	600	2,77815125	200	2,301029996
50 °C	2 h	BC DSM 4312	1400	3,146128036	11000	4,041392685
50 °C	2 h	control	25	1,397940009	25	1,397940009
50 °C	2.5 h	BC CCM 869		#ČÍSLO!	150	2,176091259
50 °C	2.5 h	BC DSM 4312		#ČÍSLO!	8600	3,934498451
50 °C	2.5 h	control		#ČÍSLO!	25	1,397940009
50 °C	3 h	BC CCM 869	1200	3,079181246	100	2
50 °C	3 h	BC DSM 4312	1300	3,113943352	10000	4
50 °C	3 h	control	25	1,397940009	25	1,397940009
50 °C	4 h	BC CCM 869	950	2,977723605	150	2,176091259
50 °C	4 h	BC DSM 4312	590	2,770852012	11000	4,041392685
50 °C	4 h	control	25	1,397940009	25	1,397940009
60 °C	0 h	BC CCM 869	1000	3	100	2
60 °C	0 h	BC DSM 4312	4700	3,672097858	16000	4,204119983
60 °C	0 h	control	25	1,397940009	25	1,397940009
60 °C	0.5 h	BC CCM 869	590	2,770852012		#ČÍSLO!
60 °C	0.5 h	BC DSM 4312	4200	3,62324929		#ČÍSLO!
60 °C	0.5 h	control	25	1,397940009		#ČÍSLO!
60 °C	1 h	BC CCM 869	1100	3,041392685	150	2,176091259
60 °C	1 h	BC DSM 4312	4300	3,633468456	6300	3,799340549
60 °C	1 h	control	25	1,397940009	25	1,397940009
60 °C	2 h	BC CCM 869	950	2,977723605	200	2,301029996
60 °C	2 h	BC DSM 4312	3900	3,591064607	6600	3,819543936
60 °C	2 h	control	25	1,397940009	25	1,397940009
60 °C	2.5 h	BC CCM 869		#ČÍSLO!	200	2,301029996
60 °C	2.5 h	BC DSM 4312		#ČÍSLO!	7000	3,84509804
60 °C	2.5 h	control		#ČÍSLO!	25	1,397940009
60 °C	3 h	BC CCM 869	640	2,806179974	200	2,301029996
60 °C	3 h	BC DSM 4312	5200	3,716003344	6600	3,819543936
60 °C	3 h	control	25	1,397940009	25	1,397940009
60 °C	4 h	BC CCM 869	1100	3,041392685	150	2,176091259
60 °C	4 h	BC DSM 4312	4600	3,662757832	6800	3,832508913
60 °C	4 h	control	25	1,397940009	25	1,397940009

Tomato sauce						
Temp	Hour	Strain	experiment	log CFU/g	experiment 2	log CFU/g
40 °C	0 h	BC CCM 869	1200	3,079181246	400	2,602059991
40 °C	0 h	BC DSM 4312	25	1,397940009	680	2,832508913
40 °C	0 h	control	25	1,397940009	25	1,397940009
40 °C	0,5 h	BC CCM 869	1500	3,176091259		#ČÍSLO!
40 °C	0,5 h	BC DSM 4312	100	2		#ČÍSLO!
40 °C	0,5 h	control	25	1,397940009		#ČÍSLO!
40 °C	1 h	BC CCM 869	1200	3,079181246	150	2,176091259
40 °C	1 h	BC DSM 4312	25	1,397940009	950	2,977723605
40 °C	1 h	control	25	1,397940009	25	1,397940009
40 °C	2 h	BC CCM 869	950	2,977723605	50	1,698970004
40 °C	2 h	BC DSM 4312	100	2	860	2,934498451
40 °C	2 h	control	25	1,397940009	25	1,397940009
40 °C	2,5 h	BC CCM 869		#ČÍSLO!	150	2,176091259
40 °C	2,5 h	BC DSM 4312		#ČÍSLO!	860	2,934498451
40 °C	2,5 h	control		#ČÍSLO!	25	1,397940009
40 °C	3 h	BC CCM 869	1100	3,041392685	150	2,176091259
40 °C	3 h	BC DSM 4312	25	1,397940009	1000	3
40 °C	3 h	control	25	1,397940009	25	1,397940009
40 °C	4 h	BC CCM 869	1200	3,079181246	350	2,544068044
40 °C	4 h	BC DSM 4312	50	1,698970004	950	2,977723605
40 °C	4 h	control	25	1,397940009	25	1,397940009
50 °C	0 h	BC CCM 869	1000	3	25	1,397940009
50 °C	0 h	BC DSM 4312	350	2,544068044	150	2,176091259
50 °C	0 h	control	25	1,397940009	25	1,397940009
50 °C	0,5 h	BC CCM 869	1300	3,113943352		#ČÍSLO!
50 °C	0,5 h	BC DSM 4312	300	2,477121255		#ČÍSLO!
50 °C	0,5 h	control	25	1,397940009		#ČÍSLO!
50 °C	1 h	BC CCM 869	1000	3	100	2
50 °C	1 h	BC DSM 4312	250	2,397940009	250	2,397940009
50 °C	1 h	control	25	1,397940009	25	1,397940009
50 °C	2 h	BC CCM 869	800	2,903089987	25	1,397940009
50 °C	2 h	BC DSM 4312	860	2,934498451	350	2,544068044
50 °C	2 h	control	25	1,397940009	25	1,397940009
50 °C	2,5 h	BC CCM 869		#ČÍSLO!	25	1,397940009
50 °C	2,5 h	BC DSM 4312		#ČÍSLO!	350	2,544068044
50 °C	2,5 h	control		#ČÍSLO!	25	1,397940009
50 °C	3 h	BC CCM 869	770	2,886490725	25	1,397940009
50 °C	3 h	BC DSM 4312	200	2,301029996	400	2,602059991
50 °C	3 h	control	25	1,397940009	25	1,397940009
50 °C	4 h	BC CCM 869	1000	3	25	1,397940009
50 °C	4 h	BC DSM 4312	25	1,397940009	100	2
50 °C	4 h	control	25	1,397940009	25	1,397940009
60 °C	0 h	BC CCM 869	1100	3,041392685	150	2,176091259
60 °C	0 h	BC DSM 4312	150	2,176091259	400	2,602059991
60 °C	0 h	control	25	1,397940009	25	1,397940009
60 °C	0,5 h	BC CCM 869	1000	3		#ČÍSLO!
60 °C	0,5 h	BC DSM 4312	400	2,602059991		#ČÍSLO!
60 °C	0,5 h	control	25	1,397940009		#ČÍSLO!
60 °C	1 h	BC CCM 869	1000	3	50	1,698970004
60 °C	1 h	BC DSM 4312	50	1,698970004	500	2,698970004
60 °C	1 h	control	25	1,397940009	25	1,397940009
60 °C	2 h	BC CCM 869	590	2,770852012	25	1,397940009
60 °C	2 h	BC DSM 4312	300	2,477121255	590	2,770852012
60 °C	2 h	control	25	1,397940009	25	1,397940009
60 °C	2,5 h	BC CCM 869		#ČÍSLO!	50	1,698970004
60 °C	2,5 h	BC DSM 4312		#ČÍSLO!	50	1,698970004
60 °C	2,5 h	control		#ČÍSLO!	25	1,397940009
60 °C	3 h	BC CCM 869	450	2,653212514	25	1,397940009
60 °C	3 h	BC DSM 4312	550	2,740362689	100	2
60 °C	3 h	control	25	1,397940009	25	1,397940009
60 °C	4 h	BC CCM 869	400	2,602059991	25	1,397940009
60 °C	4 h	BC DSM 4312	25	1,397940009	350	2,544068044
60 °C	4 h	control	25	1,397940009	25	1,397940009

Cooked rice						
Temp	Hour	Strain	experiment	log CFU/g	experiment	log CFU/g
40 °C	0 h	BC CCM 869	350	2,544068044	200	2,301029996
40 °C	0 h	BC DSM 4312	2400	3,380211242	300	2,477121255
40 °C	0 h	control	25	1,397940009	25	1,397940009
40 °C	0,5 h	BC CCM 869	350	2,544068044		#ČÍSLO!
40 °C	0,5 h	BC DSM 4312	3000	3,477121255		#ČÍSLO!
40 °C	0,5 h	control	25	1,397940009		#ČÍSLO!
40 °C	1 h	BC CCM 869	100	2	150	2,176091259
40 °C	1 h	BC DSM 4312	3300	3,51851394	400	2,602059991
40 °C	1 h	control	25	1,397940009	25	1,397940009
40 °C	2 h	BC CCM 869	200	2,301029996	25	1,397940009
40 °C	2 h	BC DSM 4312	7200	3,857332496	1100	3,041392685
40 °C	2 h	control	25	1,397940009	25	1,397940009
40 °C	2,5 h	BC CCM 869		#ČÍSLO!	25	1,397940009
40 °C	2,5 h	BC DSM 4312		#ČÍSLO!	2000	3,301029996
40 °C	2,5 h	control		#ČÍSLO!	25	1,397940009
40 °C	3 h	BC CCM 869	500	2,698970004	25	1,397940009
40 °C	3 h	BC DSM 4312	120000	5,079181246	7000	3,84509804
40 °C	3 h	control	25	1,397940009	25	1,397940009
40 °C	4 h	BC CCM 869	590	2,770852012	25	1,397940009
40 °C	4 h	BC DSM 4312	3000000	6,477121255	370000	5,568201724
40 °C	4 h	control	350	2,544068044	25	1,397940009
50 °C	0 h	BC CCM 869	150	2,176091259	25	1,397940009
50 °C	0 h	BC DSM 4312	2200	3,342422681	250	2,397940009
50 °C	0 h	control	25	1,397940009	25	1,397940009
50 °C	0,5 h	BC CCM 869	50	1,698970004		#ČÍSLO!
50 °C	0,5 h	BC DSM 4312	1700	3,230448921		#ČÍSLO!
50 °C	0,5 h	control	25	1,397940009		#ČÍSLO!
50 °C	1 h	BC CCM 869	300	2,477121255	100	2
50 °C	1 h	BC DSM 4312	2900	3,462397998	150	2,176091259
50 °C	1 h	control	25	1,397940009	25	1,397940009
50 °C	2 h	BC CCM 869	200	2,301029996	25	1,397940009
50 °C	2 h	BC DSM 4312	1700	3,230448921	150	2,176091259
50 °C	2 h	control	25	1,397940009	25	1,397940009
50 °C	2,5 h	BC CCM 869		#ČÍSLO!	50	1,698970004
50 °C	2,5 h	BC DSM 4312		#ČÍSLO!	450	2,653212514
50 °C	2,5 h	control		#ČÍSLO!	25	1,397940009
50 °C	3 h	BC CCM 869	50	1,698970004	50	1,698970004
50 °C	3 h	BC DSM 4312	1900	3,278753601	400	2,602059991
50 °C	3 h	control	25	1,397940009	25	1,397940009
50 °C	4 h	BC CCM 869	300	2,477121255	25	1,397940009
50 °C	4 h	BC DSM 4312	2200	3,342422681	25	1,397940009
50 °C	4 h	control	25	1,397940009	25	1,397940009
60 °C	0 h	BC CCM 869	150	2,176091259	100	2
60 °C	0 h	BC DSM 4312	1400	3,146128036	150	2,176091259
60 °C	0 h	control	25	1,397940009	25	1,397940009
60 °C	0,5 h	BC CCM 869	200	2,301029996		#ČÍSLO!
60 °C	0,5 h	BC DSM 4312	1600	3,204119983		#ČÍSLO!
60 °C	0,5 h	control	25	1,397940009		#ČÍSLO!
60 °C	1 h	BC CCM 869	300	2,477121255	100	2
60 °C	1 h	BC DSM 4312	1400	3,146128036	25	1,397940009
60 °C	1 h	control	25	1,397940009	25	1,397940009
60 °C	2 h	BC CCM 869	150	2,176091259	50	1,698970004
60 °C	2 h	BC DSM 4312	3300	3,51851394	100	2
60 °C	2 h	control	25	1,397940009	25	1,397940009
60 °C	2,5 h	BC CCM 869		#ČÍSLO!	250	2,397940009
60 °C	2,5 h	BC DSM 4312		#ČÍSLO!	100	2
60 °C	2,5 h	control		#ČÍSLO!	25	1,397940009
60 °C	3 h	BC CCM 869	350	2,544068044	50	1,698970004
60 °C	3 h	BC DSM 4312	1600	3,204119983	25	1,397940009
60 °C	3 h	control	25	1,397940009	25	1,397940009
60 °C	4 h	BC CCM 869	680	2,832508913	25	1,397940009
60 °C	4 h	BC DSM 4312	2600	3,414973348	25	1,397940009
60 °C	4 h	control	25	1,397940009	25	1,397940009

Mashed potatoes						
Temp	Hour	Strain	experiment	log CFU/g	experiment	log CFU/g
40 °C	0 h	BC CCM 869	900	2,954242509	1200	3,079181246
40 °C	0 h	BC DSM 4312	3200	3,505149978	14000	4,146128036
40 °C	0 h	control	25	1,397940009	25	1,397940009
40 °C	0,5 h	BC CCM 869	1100	3,041392685		#ČÍSLO!
40 °C	0,5 h	BC DSM 4312	2900	3,462397998		#ČÍSLO!
40 °C	0,5 h	control	25	1,397940009		#ČÍSLO!
40 °C	1 h	BC CCM 869	1500	3,176091259	1100	3,041392685
40 °C	1 h	BC DSM 4312	3100	3,491361694	11000	4,041392685
40 °C	1 h	control	25	1,397940009	25	1,397940009
40 °C	2 h	BC CCM 869	1100	3,041392685	950	2,977723605
40 °C	2 h	BC DSM 4312	3400	3,531478917	8600	3,934498451
40 °C	2 h	control	50	1,698970004	25	1,397940009
40 °C	2,5 h	BC CCM 869		#ČÍSLO!	1000	3
40 °C	2,5 h	BC DSM 4312		#ČÍSLO!	17000	4,230448921
40 °C	2,5 h	control		#ČÍSLO!	300	2,477121255
40 °C	3 h	BC CCM 869	1300	3,113943352	1000	3
40 °C	3 h	BC DSM 4312	84000	4,924279286	43000	4,633468456
40 °C	3 h	control	19000	4,278753601	200	2,301029996
40 °C	4 h	BC CCM 869	320000	5,505149978	4100	3,612783857
40 °C	4 h	BC DSM 4312	910000	5,959041392	7200000	6,857332496
40 °C	4 h	control	3300	3,51851394	24000	4,380211242
50 °C	0 h	BC CCM 869	25	1,397940009	730	2,86332286
50 °C	0 h	BC DSM 4312	5600	3,748188027	17000	4,230448921
50 °C	0 h	control	25	1,397940009	25	1,397940009
50 °C	0,5 h	BC CCM 869	25	1,397940009		#ČÍSLO!
50 °C	0,5 h	BC DSM 4312	5300	3,72427587		#ČÍSLO!
50 °C	0,5 h	control	25	1,397940009		#ČÍSLO!
50 °C	1 h	BC CCM 869	25	1,397940009	950	2,977723605
50 °C	1 h	BC DSM 4312	6000	3,77815125	12000	4,079181246
50 °C	1 h	control	25	1,397940009	25	1,397940009
50 °C	2 h	BC CCM 869	25	1,397940009	1100	3,041392685
50 °C	2 h	BC DSM 4312	3600	3,556302501	10000	4
50 °C	2 h	control	25	1,397940009	25	1,397940009
50 °C	2,5 h	BC CCM 869		#ČÍSLO!	860	2,934498451
50 °C	2,5 h	BC DSM 4312		#ČÍSLO!	11000	4,041392685
50 °C	2,5 h	control		#ČÍSLO!	25	1,397940009
50 °C	3 h	BC CCM 869	25	1,397940009	910	2,959041392
50 °C	3 h	BC DSM 4312		#ČÍSLO!	10000	4
50 °C	3 h	control	25	1,397940009	25	1,397940009
50 °C	4 h	BC CCM 869	25	1,397940009	860	2,934498451
50 °C	4 h	BC DSM 4312	2500	3,397940009	12000	4,079181246
50 °C	4 h	control	25	1,397940009	25	1,397940009
60 °C	0 h	BC CCM 869	25	1,397940009	770	2,886490725
60 °C	0 h	BC DSM 4312	3200	3,505149978	17000	4,230448921
60 °C	0 h	control	25	1,397940009	25	1,397940009
60 °C	0,5 h	BC CCM 869	25	1,397940009		#ČÍSLO!
60 °C	0,5 h	BC DSM 4312	2600	3,414973348		#ČÍSLO!
60 °C	0,5 h	control	25	1,397940009		#ČÍSLO!
60 °C	1 h	BC CCM 869	25	1,397940009	1200	3,079181246
60 °C	1 h	BC DSM 4312	2500	3,397940009	4700	3,672097858
60 °C	1 h	control	25	1,397940009	25	1,397940009
60 °C	2 h	BC CCM 869	25	1,397940009	1400	3,146128036
60 °C	2 h	BC DSM 4312	2700	3,431363764	5000	3,698970004
60 °C	2 h	control	25	1,397940009	25	1,397940009
60 °C	2,5 h	BC CCM 869		#ČÍSLO!	1100	3,041392685
60 °C	2,5 h	BC DSM 4312		#ČÍSLO!	14000	4,146128036
60 °C	2,5 h	control		#ČÍSLO!	25	1,397940009
60 °C	3 h	BC CCM 869	25	1,397940009	1400	3,146128036
60 °C	3 h	BC DSM 4312		#ČÍSLO!	6800	3,832508913
60 °C	3 h	control	25	1,397940009	25	1,397940009
60 °C	4 h	BC CCM 869	25	1,397940009	1000	3
60 °C	4 h	BC DSM 4312	3000	3,477121255	7300	3,86332286
60 °C	4 h	control	25	1,397940009	25	1,397940009
